# Comparative accuracy of typhoid diagnostic tools: A Bayesian latent-class network analysis

**DOI:** 10.1371/journal.pntd.0007303

**Published:** 2019-05-08

**Authors:** Paul Arora, Kristian Thorlund, Darren R. Brenner, Jason R. Andrews

**Affiliations:** 1 Dalla Lana School of Public Health, Division of Epidemiology, University of Toronto, Ontario, Canada; 2 Department of Health Research Methods, Evidence, and Impact, McMaster University, Hamilton, Ontario, Canada; 3 Departments of Oncology and Community Health Sciences, Cumming School of Medicine, University of Calgary, Calgary, Alberta, Canada; 4 Division of Infectious Diseases and Geographic Medicine, Stanford University, Stanford, California, United States of America; The University of Sheffield, UNITED KINGDOM

## Abstract

**Background:**

Typhoid fevers are infections caused by the bacteria *Salmonella enterica* serovar Typhi (*Salmonella* Typhi) and Paratyphi A, B and C (*Salmonella* Paratyphi). Approximately 17.8 million incident cases of typhoid fever occur annually, and incidence is highest in children. The accuracy of current diagnostic tests of typhoid fever is poorly understood. We aimed to determine the comparative accuracy of available tests for the pediatric population.

**Methods:**

We first conducted a systematic literature review to identify studies that compared diagnostic tests for typhoid fever in children (aged ≤15 years) to blood culture results. We applied a Bayesian latent-class extension to a network meta-analysis model. We modelled known diagnostic properties of bone marrow culture and the relationship between bone marrow and blood culture as informative priors in a Bayesian framework. We tested sensitivities for the proportion of negative blood samples that were false as well as bone marrow sensitivity and specificity.

**Results:**

We found 510 comparisons from 196 studies and 57 specific to the pediatric population. IgM-based tests outperformed their IgG-based counterparts for ELISA and Typhidot tests. The lateral flow IgG test performed comparatively well with 92% sensitivity (72% to 98% across scenario analyses) and 94% specificity. The most sensitive test of those investigated for the South Asian pediatric population was the Reverse Passive Hemagglutination Assay with 99% sensitivity (98% - 100% across scenario analyses). Adding a Widal slide test to other typhoid diagnostics did not substantially improve diagnostic performance beyond the single test alone, however, a lateral flow-based IgG rapid test combined with the typhoid/paratyphoid (TPT) assay yielded improvements in sensitivity without substantial declines in specificity and was the best performing combination test in this setting.

**Conclusion:**

In the pediatric population, lateral-flow IgG, TPT and Reverse Passive Hemagglutination tests had high diagnostic accuracy compared to other diagnostics. Combinations of tests may provide a feasible option to increase diagnostic sensitivity. South Asia has the most informed set of data on typhoid diagnostic testing accuracy, and the evidence base in other important regions needs to be expanded.

## Author summary

Typhoid fever is an infection caused by the bacterium *Salmonella* Typhi. Typhoid fever is rare in developed countries but remains high in the developing world. Effective treatment is available but accurate diagnosis of typhoid fever is challenging as typhoid fever can be difficult to distinguish from other infections. Bone marrow culture is the most accurate diagnostic test for typhoid fever however is invasive and not feasible in many settings. New vaccines for typhoid and the need for improved estimates of burden increases the demand for improved understanding of diagnostic accuracy. Comparing the diagnostic accuracy of tests for typhoid fever is challenging as head-to-head studies are few. We applied newly developed methods for comparative evaluation of diagnostic tests for typhoid fever in children using statistical approaches that allowed for the proper incorporation of uncertainty and comparison of tests that had not been compared directly. The lateral-flow IgG, TPT and Reverse Passive Hemagglutination tests all had good diagnostic accuracy compared to other diagnostics. Combinations of tests may provide a feasible option to increase diagnostic sensitivity. Finally, while South Asia has the most informed set of data on typhoid diagnostic testing accuracy, the evidence base in other important regions needs to be expanded.

## Introduction

Typhoid fever (also known as enteric fever) is a systemic infection caused by the Gram-negative bacteria *Salmonella enterica* serotypes Typhi or Paratyphi A,B and C[1]^,^[2]. While rare in developed countries, the burden of typhoid remains high in developing countries. Recent annual estimates of typhoid fever cases in low- and middle-income countries range from approximately 17.8 million[3] to 26.9 million[4] cases worldwide and most of these are in South Asia. The pediatric population is of particular interest as most cases occur in those between 3 and 19 years of age[1], the highest incidence of typhoid occurs in those less than 5 years of age[5]. Recent modelling work reported a higher incidence among children aged two to four years compared to those less than two years.[3] With the recent World Health Organization pre-qualification of, and GAVI commitments towards, a typhoid conjugate vaccine for use in routine immunization programs, there is a need for better data on typhoid burden in young children, which requires better understanding of diagnostic accuracy. Prior meta-analyses have focused on all age groups without distinguishing performance in children; however, we hypothesize that diagnostic accuracy may differ between children and adults due to a greater degree of prior exposure to Salmonella and other pathogens in adults, leading to serologic cross reactivity. If diagnosed promptly, typhoid can be successfully treated with antibiotics. [1, 2]

Accurate diagnosis of typhoid fever has proved a major challenge. Clinical signs and symptoms are often non-specific, and typhoid can be difficult to distinguish from other acute febrile illnesses, including dengue, malaria, influenza, leptospirosis, and Rickettsial infections[6–8]. The definitive diagnosis for typhoid fever is via isolation of *S*. Typhi from blood, bone marrow or other sterile sites.[1] The most sensitive and specific diagnostic test for typhoid fever is bone marrow culture; however, as this test is invasive, carries risks of medical complications, and requires technical expertise and specialized equipment, it is not widely performed in endemic settings as a routine diagnostic procedure. Among culture-based methods, blood culture is the most commonly used typhoid diagnostic method, but results are not available for days, and many settings lack the resources required for proper culturing techniques. Furthermore, it has limited sensitivity (40–75% in most settings)[9, 10], which may be further diminished by prior antibiotic use.

The Widal test, developed in the late 19th century to measure antibodies against the O and H antigens of *Salmonella*, remains perhaps the most widely used typhoid diagnostic in the world. However, the Widal test only has moderate sensitivity and specificity, particularly in endemic settings, and there remains a challenge of determining a proper cut-off point for a positive result[5, 11]. Indeed, rapid and reliable (>90% sensitivity and specificity) diagnostics do not yet exist for invasive salmonellosis. The Reverse Passive Hemagglutination (RPHA) Test, that detects the *S*. Typhi antigen, was found to have a sensitivity and specificity that is comparable with the Widal test leading to suggestion that it could be used as an alternative to the Widal test in busy microbiology laboratories[12, 13]. Newer diagnostic tests, such as the antibody tests Typhidot and Tubex, have demonstrated moderate accuracy[14]. The typhoid/paratyphoid diagnostic assay (TPT test) has shown promising results.[15] Polymerase chain reaction (PCR) and other molecular, transcriptomic and metabolomic methods have been developed, but they have yet to be evaluated in large scale settings.

Assessing the comparative performance of diagnostic testing is challenging as few head-to-head evaluations exist and previous reviews of diagnostic testing have found a high level of variation in testing methods for typhoid fever globally and a lack of a single applicable gold standard, a challenge that is particularly acute given the low sensitivity of the most common reference standard, blood culture.[3, 9] We aimed to assess the comparative performance of typhoid diagnostics using newly developed methods for comparative evaluations [16]. In particular, we combined a Bayesian network meta-analysis (NMA) procedure with latent class analysis. [16].

## Methods

We developed a comprehensive search strategy to identify relevant studies comparing diagnostic tests for typhoid disease. We particularly considered typhoid fever to include *Salmonella* Typhi and *S*. Paratyphi A. We searched the following databases: EMBASE, MEDLINE, ISI Web of Science and the Cochrane Central Register of Controlled Trials from inception to December 26, 2016. We also scanned references from systematic reviews on typhoid diagnostic tools identified via the above search. We conducted a grey literature search of Google Scholar and the National Institutes of Health Research Portfolio Online Reporting Tools (NIH RePORT). We searched conference proceedings of the International Conference on Typhoid and Other Invasive Salmonelloses and the American Society of Tropical Medicine and Hygiene Conference, and unpublished data submitted by the originator companies to the US Food and Drug Administration and the European Medicines Agency as part of diagnostic registration applications. Additionally, we performed manual searches of clinicaltrials.gov and the WHO International Clinical Trials Registry Platform to identify studies that have not yet been published but have results and were potentially eligible for inclusion. Specific search terms and results by database are provided in S1 Table. We also engaged key leaders from disparate agencies that conduct research in diagnostic development, including, but not limited to the U.S. Department of Defense (Walter Reed Army Institute of Research and Defense Advanced Research Projects Agency) and non-profit research institutions and diagnostic development organizations.

### Data extraction

All abstract and full-text screening of studies was done in duplicate. Data extraction was completed using a standardized data extraction form. The extraction form was designed for this study and pilot tested by the authors. A copy of our extraction form is included in S2 Table. We extracted all comparisons across diagnostic tests as well as within any relevant subgroups presented in the included studies. Study characteristics of interest for extraction included: detailed description of diagnostic tests used including the details of any commercial tests used, types and volume of biological specimen, study location (detailed location, country and coded into World Bank region), broad age group of study population, duration of illness (most often reported as duration of fever), patient reported antibiotic self-treatment/use prior to study entry. For studies where subgroup data were not reported, study authors were contacted for age-specific contingency tables. Data were analyzed at the study level and at the level of individual test comparison (index test *versus* reference test) with both test result and disease status dichotomized.

Pair-wise meta-analysis or network meta-analysis was only done in a subset of studies. This subset was in populations of children, approximately aged 15 or younger (in some cases, it was clear that most subjects were children, but we could not be certain that teenagers and those over 15 years of age were not included) that used blood culture alone as the diagnostic reference test and were conducted in one of three World Bank regions: South Asia, East Asia & Pacific (EAP) and sub-Saharan Africa. These restrictions were introduced to reduce heterogeneity across studies, make synthesis results more interpretable, and focus on pediatric cases in typhoid endemic regions.

### Statistical methods

#### Pairwise meta-analysis for diagnostic tests

To generate summary estimates of sensitivity and specificity among a subset of diagnostic test comparisons, we conducted meta-analysis for diagnostic tests using methods proposed by Reitsma et al.[17] Briefly, diagnostic accuracy is generally summarized by two measures (usually sensitivity and specificity or likelihood ratios) and these measures are correlated.[18] Because of the correlated nature of the two measures synthesis of diagnostic testing accuracy estimates requires more involved methods than standard meta-analysis applications. This is true even in our “simple” situation where comparisons from each primary study are summarized as a 2 × 2 table of test results against true disease status, both of which have been dichotomized.[18] We used the bivariate model, developed by Reitsma et al.[17], that accounted for between-study heterogeneity as well as correlation between sensitivity and specificity (further details are provided in S1 Statistical appendix).

#### Bayesian latent class network meta-analysis of diagnostic tests

To establish the comparative diagnostic accuracy between tests, diagnostic test network meta-analysis was performed. We built on the models previously proposed by Menten and Lesaffre[16], with some modifications to fit the data structure for typhoid diagnostic testing. The mathematical expressions of the model and the statistical code for the Bayesian diagnostic test network meta-analysis (programmed in OpenBUGS) are provided in S1 Statistical appendix.

Since a key limitation in typhoid diagnostic test research is the absence of a ‘gold reference standard’ across studies (i.e. bone marrow culture), conventional network meta-analysis of diagnostic test accuracy studies cannot provide comparative sensitivity and specificity estimates with respect to ‘the truth’. Rather, the most common reference test is blood culture, which is often assumed to yield in the range of 40–75% sensitivity and 100% specificity. More recent synthesis estimates have placed sensitivity estimates higher at 66% when compared to bone marrow[10]. To obtain comparative estimates of sensitivity and specificity with respect to bone marrow culture, we therefore applied a latent class extension to the conventional network meta-analysis model. The Bayesian latent class model proposed by Menten and Lesaffre[16] require good study population prevalence estimates, which was not available for typhoid disease since all studies only enrolled patient with suspected typhoid fever. Rather, we implemented known diagnostics properties of bone marrow culture and the relationship between bone marrow and blood culture as informative priors to facilitate a novel Bayesian latent class diagnostic test network meta-analysis. Particularly, it is estimated that the sensitivity of blood culture for diagnosis of typhoid is only 50–60%.[19] Thus, resampling these to become positive with a corresponding probability theoretically corresponds to a latent class gold standard. Further, applying highly informative priors on the sensitivity and specificity corresponding to that of bone marrow culture will aid in stabilizing the Bayesian model and posterior distributions converge to global maxima Markov states. Lastly, according to good Bayesian practice, use of informative priors should be subjected to sensitivity analysis, referring to different “scenarios”. We thus tested sensitivities for the proportion of negative blood samples that were false negative (base case 50%, sensitivity range 33.3% to 66.7%), as well as bone marrow sensitivity and specificity (base case 95% sensitive and 99% specific, scenario analysis 85% sensitive and 99% specific).

Because there was substantial heterogeneity in the specific types of serologic and molecular tests used, with very few studies utilizing the same antigen-isotype combinations, diagnostic platforms, or molecular targets, we aggregated diagnostic tests according to class (antibody tests, antigen tests, PCR-based tests) to present summary estimates for these diagnostic classes.

#### Estimating diagnostic accuracy of combinations of rapid tests

Since the network analysis simultaneously links the sensitivity and specificity estimates (on the logit scale) to the latent class ‘gold standard’, it is possible to estimate the diagnostic accuracy of a combination of two tests within the MCMC sampling framework from the conditionality of the posterior distributions. In particular, the sensitivity of a combination test that is considered positive if either of the two tests are positive can be represented mathematically as the maximum of the two tests within a sampling scheme of individual patient outcomes. Within the MCMC sampling scheme, this should approximately correspond to sampling of the maximum sensitivity of the two sensitivity nodes for each MCMC iteration. Likewise, the specificity of a combination of tests that is considered negative only if both tests are negative can be represented with the minimum of the two.

## Results

**Fig 1:**
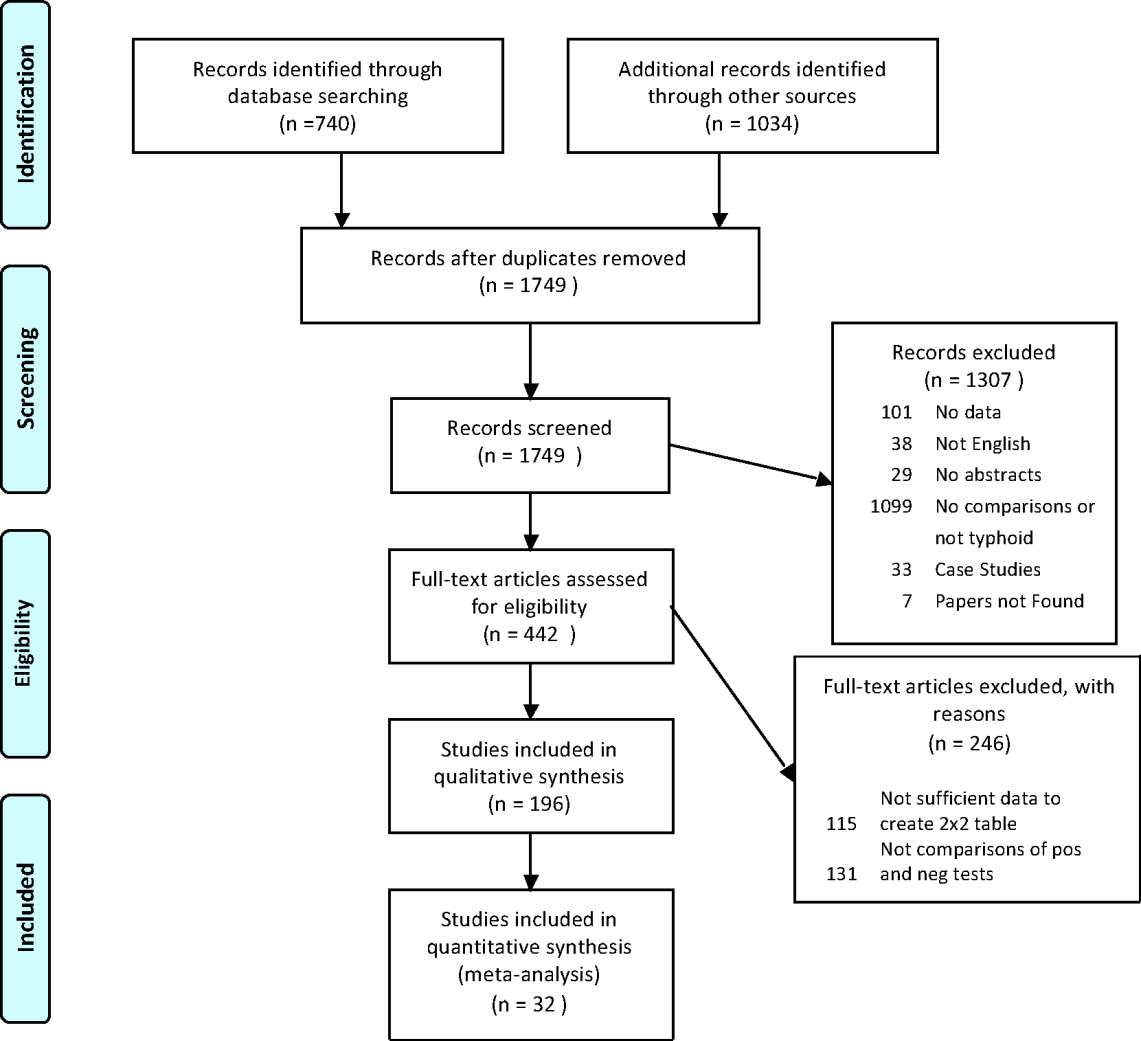
The PRISMA flow diagram for the systematic literature review of diagnostic tests for typhoid fever.

From a combined 1,749 records identified, there were 196 studies included for full-extraction (See Fig 1 for flow diagram). From these studies, 57 comparisons between tests from 32 studies were included for the NMA (studies listed in Table 1). Full datasets for study level characteristics and comparison level data are presented in S3 and S4 Tables. A glossary of terms is provided in S5 Table.

**Table 1:** Summary of population characteristics from the studies included in the systematic literature review.

Study Characteristics	Full set of studies		NMA set of studies	
	n	(%)	n	(%)
Total Studies	196		32	
**World Bank Regions**				
East Asia and Pacific	53	30.11	5	15.63
South Asia	74	42.05	23	71.88
Sub-Saharan Africa	16	9.09	4	12.50
Europe and Central Asia	6	3.41		
Latin America and Caribbean	6	3.41		
Middle East and North Africa	15	8.52		
Multiple	5	2.84		
Unknown	1	0.57		
**Patient Age Mix**				
Adult	17	9.83		
Adult/Child	62	35.84		
Child	40	23.12	32	100
Unknown/Not reported	54	31.21		
**Typhoid Endemicity**				
High	121	68.36	28	87.50
Medium	47	26.55	4	12.50
Low	5	2.82		
Mixed	3	1.69		
Unknown/Not reported	1	0.56		
**Patient Antibiotic Status**				
Mixed	20	11.56	5	15.63
No	12	6.94	1	3.13
Unknown/Not reported	125	72.25	24	75.00
Yes	16	9.25	2	6.25
Volume of Sample Collected*				
<2 ml	2	2.5	1	3.13
2-<5 ml	19	23.75	8	25.00
5-<8 ml	30	37.5	7	21.88
≥8 ml	29	36.25	3	9.38
**Year of study publication**				
Pre 1990	41	20.92	2	6.25
1990 to 1999	42	21.43	4	12.50
2000 to 2009	56	28.57	9	28.13
2010 to Present	57	29.08	17	53.13
**Duration of Symptoms (days)**				
<7	38	46.91	13	40.63
7–14	38	46.91	6	18.75
≥14	5	6.17		

*Not reported in the majority of studies

### SLR descriptive characteristics for studies and comparisons

The summary results of the search are presented in Tables 2 and 3 separated by the full set of studies and the subset of studies included the NMA. The full set of studies includes all 196 identified studies in our search that represented 510 pairwise comparisons between two typhoid diagnostic tests. The subset of 32 studies used in NMA represented 57 comparisons.

**Table 2:** Summary of diagnostic test comparisons included in the systematic literature review.

Comparison Descriptions	Full set of studies		NMA set of studies	
Index v Ref	n	(%)	n	(%)
Total Number of Comparisons	510		57	
Antibody v Clinical	1	0.2		
Antibody v Composite	25	4.9		
Antibody v Culture	164	32.2	36	63.16
			EAP: 5	
			SA: 27	
			SSA: 4	
Antibody v DNA	4	0.8		
Antibody v Widal	7	1.4		
Antigen v Composite	14	2.8		
Antigen v Culture	40	7.8	3	5.26
			EAP: 2	
			SA: 1	
			SSA: 0	
Antigen v DNA	1	0.2		
Antigen v Widal	7	1.4		
Clinical v Culture	1	0.2		
Composite v Composite	4	0.8		
Composite v Culture	4	0.8		
Culture v Clinical	6	1.2		
Culture v Composite	3	0.6		
Culture v Culture	9	1.8		
Culture v Culture/DNA	7	1.4		
Culture v DNA	2	0.4		
DNA v Clinical	5	1.0		
DNA v Composite	1	0.2		
DNA v Culture	34	6.7	7 EAP: 1 SA: 5 SSA: 1	12.28
DNA v DNA	2	0.4		
Diazo v Culture	1	0.2	1 SA: 1	1.75
Widal v Antibody	8	1.6		
Widal v Clinical	11	2.2		
Widal v Composite	13	2.6		
Widal v Culture	129	25.3	10 SA: 10	17.54
Widal v DNA	4	0.8		
Widal v Widal	3	0.6		

*All Cultures in network meta-analysis set were from blood

EAP: East-Asia and Pacific; SA: South Asia; SSA: Sub-Saharan Africa; Clinical: refers to diagnosis based on clinical examination only (no laboratory test); Composite refers to diagnosis based on some combination of laboratory test and clinical examination. Antibody test involves the detection of antibodies in patient sample; Antigen test refers to the detection of typhoidal *Salmonella* antigens in patient sample.

**Table 3:** Pair-wise meta-analysis summary estimates of diagnostic test accuracy compared to blood culture from studies in child populations, by world bank regions.

Index test	Region	# of studies	Sensitivity	95% Lower confidence limit	95% Upper confidence limit	Specificity	95% Lower confidence limit	95% Upper confidence limit
Antibody (ELISA, IgG)	East-Asia and Pacific	2	47%	40%	55%	52%	49%	55%
Antibody (ELISA, IgM)	East-Asia and Pacific	2	63%	57%	69%	76%	74%	79%
Antibody (Lateral flow, IgM)	East-Asia and Pacific	1	55%	39%	70%	98%	96%	99%
Antigen (Rapid diagnostic test (RDT))	East-Asia and Pacific	1	91%	59%	100%	96%	81%	100%
Antigen (TUBEX TP, O12)	East-Asia and Pacific	1	100%	88%	100%	100%	93%	100%
PCR/DNA	East-Asia and Pacific	1	41%	26%	57%	100%	99%	100%
Antibody (ELISA, IgA)	South Asia	2	91%	79%	98%	79%	70%	86%
Antibody (ELISA, IgG or IgM)	South Asia	1	74%	56%	87%	80%	74%	85%
Antibody (ELISA, IgG)	South Asia	2	59%	45%	72%	71%	65%	77%
Antibody (ELISA, IgM)	South Asia	2	75%	62%	86%	78%	72%	83%
Antibody (Enterocheck WB, IgM)	South Asia	1	85%	73%	94%	89%	85%	92%
Antibody (Lateral flow, IgG)	South Asia	1	98%	90%	100%	78%	68%	86%
Antibody (Lateral flow, IgM or IgG)	South Asia	1	69%	57%	79%	71%	65%	77%
Antibody (TPT test, *S*. Typhi specific IgA)	South Asia	2	100%	94%	100%	69%	62%	75%
Antibody (TUBEX, IgM)	South Asia	4	63%	55%	71%	84%	81%	88%
Antibody (Typhidot, IgG)	South Asia	2	13%	4%	30%	33%	26%	41%
Antibody (Typhidot, IgM or IgG)	South Asia	1	65%	44%	83%	66%	50%	80%
Antibody (Typhidot, IgM)	South Asia	6	77%	70%	83%	60%	56%	65%
Antigen (Reverse Passive Hemagluttination)	South Asia	1	100%	93%	100%	76%	66%	84%
Diazo	South Asia	1	87%	69%	96%	86%	77%	93%
nested PCR (blood)	South Asia	3	45%	34%	55%	83%	78%	88%
PCR/DNA	South Asia	2	48%	27%	69%	84%	77%	90%
Widal (H) 1:160 (H)	South Asia	1	30%	15%	49%	98%	89%	100%
Widal (H) 1:200 (H), 1:100 (O)	South Asia	1	30%	15%	49%	91%	83%	96%
Widal (O or H) 1:80 (O or H)	South Asia	1	92%	75%	99%	100%	29%	100%
Widal (O) 1:160 (O)	South Asia	1	70%	51%	85%	94%	83%	99%
Widal (O) 1:180 (O)	South Asia	1	63%	51%	73%	37%	31%	43%
Widal (O) 1:80 (O)	South Asia	2	57%	49%	65%	74%	66%	80%
Widal slide (H) 1:160 (H)	South Asia	1	86%	79%	91%	98%	93%	100%
Widal slide (O or H) 1:160 (O or H)	South Asia	3	74%	65%	81%	68%	62%	74%
Widal slide (O) 1:80 (O)	South Asia	1	71%	63%	78%	98%	93%	100%
Antibody (ELISA, total Ig)	Sub-Saharan Africa	1	87%	74%	95%	75%	48%	93%
Antibody (TUBEX, IgM)	Sub-Saharan Africa	1	79%	61%	91%	89%	81%	94%
PCR/DNA	Sub-Saharan Africa	1	88%	64%	99%	86%	80%	90%
Widal (H) 1:80 (H)	Sub-Saharan Africa	1	75%	48%	93%	95%	91%	98%
Widal (O) 1:80 (O)	Sub-Saharan Africa	1	69%	41%	89%	96%	91%	98%

Study level characteristics for 196 included studies are presented in S3 Table and summarized in Table 2. Among the full set of studies, the majority were conducted in areas of high typhoid endemicity (68.4%), and 72.4% of studies were conducted in either South or East Asia (World Bank Regions classification). There was a relatively even distribution of patient age mixes between adults and children in the studies. However, many studies did not report age, and among the 62 studies that included both adults and children, no subgroup results were reported by age. Just over half of the studies (60.4%) included less than 200 patients with few studies containing more than 1000 patients. There was a slightly higher proportion of newer (post 2000) studies in the full dataset with the majority of studies in the network analysis set being conducted in 2010 or later. In both the full set of studies and the network, the majority of studies (59.2%) did not provide details on the volume of biological specimen collected for the tests or the duration of symptoms (58.7%). Prior antibiotic use can greatly influence the sensitivity of blood culture; however, 72.3% of studies did not report on this characteristic. For those studies that did provide these data we have presented these in Table 2.

### Network of evidence

**Fig 2:**
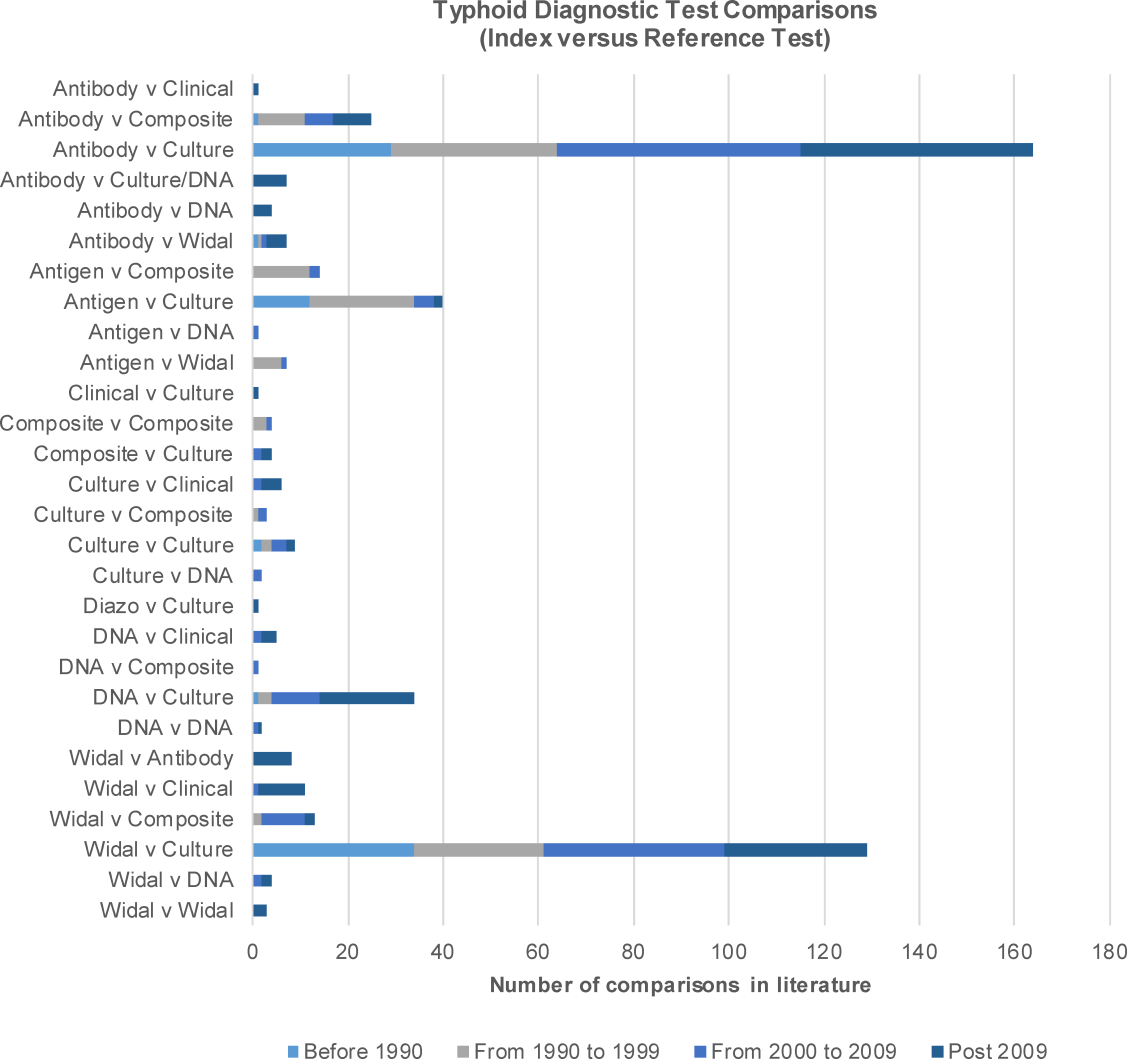
The number of comparisons for each combination of diagnostic test of typhoid fever identified in the systematic literature review.

**Fig 3:**
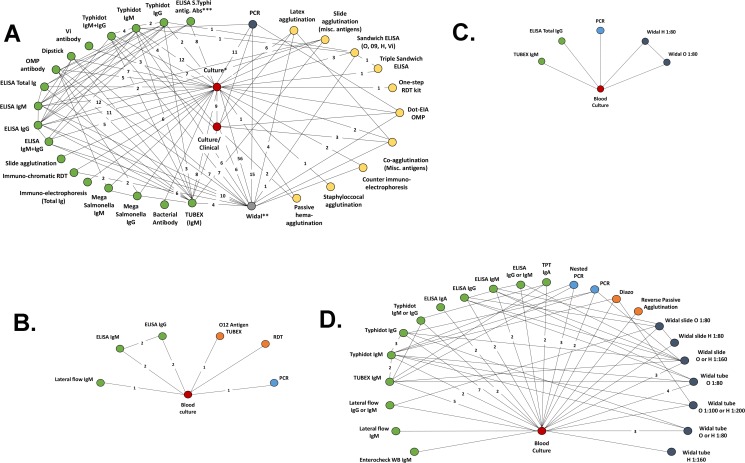
a-d. The Network of Comparisons for each Combination of Diagnostic Test of Typhoid Fever Identified in the Systematic Literature Review in a) all regions, b) East-Asia and Pacific, c) Sub-Saharan Africa and d) South Asia Footnotes: * Covers any 100% specific culture (blood, urine, bone marrow, “mix”. Analytically these will be treated as different tests. ** Covers tests for O, H and Vi antigens; titers ranging from 1:20, 1:40…1:320, 1:640 and “slide Widal”. *** Covers multiple S. Typhi antigens (also has 8 connections to Widal tests) “OMP antibody” and “Vi antibody” refers to either IgG or IgM results combined as most studies either did not report results separately by antibody class or reported them together.

Pairwise, summary estimates for meta-analysis of testing characteristics are presented in Table 3 and as forest plots in S1–S4 Figs. For our network, the numbers of comparisons across each of the six types of index and reference diagnostic tests categorized by date of publication is presented in Fig 2 and summarized in Table 4. The most common comparisons in the full set of 510 comparisons were index tests using antibody, Widal and molecular diagnostics contrasted to viable bacteria culture tests. While the Widal test is the most widely used diagnostic test for typhoid in endemic regions, the majority of the literature focused on evaluating the performance of other antibody tests. The graphical network of comparisons with the NMA set across all index and reference tests for is presented in a network structure in Fig 3A.

**Table 4:** List of studies included in the network meta-analysis.

Region	First Author	Year	Index Test(s)	Ref.
East Asia and Pacific	Castonguay -Vanier J	2013	Antigen (RDT)	[20]
East-Asia and Pacific	Handojo I	2000	Antibody (ELISA, IgG)	[21]
			Antibody (ELISA, IgM)	
East-Asia and Pacific	Limpitikul W	2014	Antibody (ELISA, IgG)	[22]
			Antibody (ELISA, IgM)	
East-Asia and Pacific	Moore CE	2014	Antibody (Lateral flow, IgM)	[23]
			PCR/DNA	
East-Asia and Pacific	Nugraha J	2012	Antigen (TUBEX TP, O12)	[24]
South Asia	Alam AS	2011	Widal slide (O or H) 1:160 (O or H)	[25]
South Asia	Ambati SR	2007	nested PCR (blood)	[26]
South Asia	Anusha R	2007	Antibody (Enteroheck WB, IgM)	[27]
South Asia	Beig FK	2010	Antibody (Typhidot, IgM)	[28]
			Diazo	
			Widal (H) 1:200 (H)	
South Asia	Das S	2013	Antibody (Lateral flow, IgM or IgG)	[29]
			Widal (O) 1:180 (O)	
South Asia	Dutta S	2006	Antibody (TUBEX, IgM)	[30]
			Antibody (Typhidot, IgM)	
			Widal (O)	
South Asia	Islam K	2016	Antibody (TPT test, S.Typhi specific IgA)	[31]
			Antibody (TUBEX, IgM)	
			Antibody (Typhidot, IgM or IgG)	
South Asia	Kalhan R	1998	Antigen (Reverse Passive Hemagluttination)	[12]
South Asia	Khan IH	2016	Antibody (Lateral flow, IgG)	[32]
South Asia	Khanam F	2013	Antibody (TPT test, S.Typhi specific IgA)	[33]
South Asia	Kulkarni ML	1994	Widal (H) 1:160 (H)	[34]
			Widal (O) 1:160 (O)	
South Asia	Kumar KS	2016	Antibody (Typhidot, IgG)	[35]
			Antibody (Typhidot, IgM)	
South Asia	Narayanappa D	2010	Antibody (Typhidot, IgM)	[36]
South Asia	Nizami SQ	2006	Antibody (TUBEX, IgM)	[37]
			Antibody (Typhidot, IgM)	
			nested PCR (blood)	
South Asia	Prakash P	2005	nested PCR (blood)	[38]
South Asia	Prakash P	2007	Antibody (Typhidot, IgG) Widal slide (O or H) 1:160 (O or H)	[39]
			Antibody (Typhidot, IgM)	
			PCR/DNA	
South Asia	Rahman M	2007	Antibody (ELISA, IgG or IgM)	[40]
			Antibody (ELISA, IgG)	
			Antibody (ELISA, IgM)	
			Antibody (TUBEX, IgM)	
			Widal slide (O or H) 1:160 (O or H)	
South Asia	Saha SK	1996	Widal slide (H) 1:160 (H)	[41]
			Widal slide (O) 1:80 (O)	
South Asia	Shehabi AA	1981	Widal (O or H) 1:80 (O or H)	[42]
South Asia	Sheikh A	2009	Antibody (ELISA, IgA)	[43]
South Asia	Srivastava L	1986	Antibody (ELISA, IgG)	[44]
			Antibody (ELISA, IgM)	
			Widal (O) 1:80 (O)	
South Asia	Tennant SM	2015	PCR/DNA	[45]
South Asia	Zaka-ur-Rab Z	2012	Antibody (ELISA, IgA)	[46]
Sub-Saharan Africa	Al-Emran HM	2016	PCR/DNA	[47]
Sub-Saharan Africa	Cheesbrough JS	1997	Antibody (ELISA, total Ig)	[48]
Sub-Saharan Africa	Ley B	2010	Widal (H) 1:80 (H)	[49]
			Widal (O) 1:80 (O)	
Sub-Saharan Africa	Ley B	2011	Antibody (TUBEX, IgM)	[50]

### Comparative sensitivity and specificity from Bayesian latent class network meta-analysis

A network of evidence was generated overall (Fig 3A) and for each World Bank Region under study (Fig 3B–3D). The testing characteristics generated from Bayesian analysis are presented in Tables 5–8.

**Table 5:** Results from Bayesian latent class network meta-analysis in all regions. Sensitivity and specificity in pediatric patients compared with a blood culture reference test or theoretical bone marrow culture test.

	Diagnostic accuracy against blood culture		Diagnostic accuracy against latent class bone marrow culture	
Test	Sensitivity (95%CI)	Specificity (95%CI)	Sensitivity (Scenario min-max)	Specificity (scenario min-max)
Antibody (Enterocheck WB, IgM)	85% (73% - 94%)	73% (94% - 89%)	39% (30% - 53%)	97% (96% - 97%)
Antibody (ELISA, IgA)	91% (79% - 98%)	79% (98% - 79%)	64% (51% - 78%)	99% (99% - 99%)
Antibody (ELISA, IgG)	50% (44% - 57%)	44% (57% - 56%)	53% (46% - 56%)	85% (80% - 85%)
Antibody (ELISA, IgM)	66% (60% - 71%)	60% (71% - 77%)	62% (54% - 70%)	98% (96% - 98%)
Antibody (ELISA, IgG or IgM)	74% (56% - 87%)	56% (87% - 80%)	47% (37% - 58%)	93% (91% - 93%)
Antibody (ELISA, total Ig)	87% (74% - 95%)	74% (95% - 75%)	86% (84% - 88%)	100% (99% - 100%)
Antibody (Lateral flow, IgG)	98% (90% - 100%)	90% (100% - 78%)	93% (72% - 98%)	93% (92% - 94%)
Antibody (Lateral flow, IgM or IgG)	69% (57% - 79%)	57% (79% - 71%)	59% (52% - 65%)	93% (91% - 94%)
Antibody (Lateral flow, IgM)	55% (39% - 70%)	39% (70% - 98%)	13% (11% - 17%)	99% (99% - 99%)
Antibody (TUBEX, IgM)	66% (59% - 73%)	59% (73% - 85%)	44% (35% - 58%)	98% (98% - 98%)
Antibody (Typhidot, IgG)	13% (4% - 30%)	4% (30% - 33%)	37% (23% - 52%)	73% (72% - 78%)
Antibody (Typhidot, IgM)	77% (70% - 83%)	70% (83% - 60%)	80% (70% - 85%)	95% (92% - 96%)
Antigen (RDT)	91% (59% - 100%)	59% (100% - 96%)	55% (42% - 75%)	95% (94% - 96%)
Antibody (Typhidot, IgM or IgG)	65% (44% - 83%)	44% (83% - 66%)	91% (86% - 93%)	86% (86% - 87%)
Antigen (Reverse Passive Hemagluttination)	100% (93% - 100%)	93% (100% - 76%)	99% (72% - 100%)	92% (91% - 93%)
Antigen (TUBEX TP, O12)	100% (88% - 100%)	88% (100% - 100%)	77% (55% - 100%)	99% (99% - 99%)
Diazo	87% (69% - 96%)	69% (96% - 86%)	56% (46% - 74%)	94% (92% - 94%)
nested PCR (blood)	45% (34% - 55%)	34% (55% - 83%)	39% (36% - 43%)	94% (94% - 94%)
PCR/DNA	52% (41% - 63%)	41% (63% - 94%)	25% (21% - 34%)	99% (99% - 99%)
Widal slide (O) 1:80 (O)	71% (63% - 78%)	63% (78% - 98%)	60% (53% - 66%)	98% (98% - 98%)
Widal slide (H) 1:160 (H)	86% (79% - 91%)	79% (91% - 98%)	79% (69% - 82%)	98% (98% - 99%)
Widal slide (O or H) 1:160 (O or H)	71% (60% - 80%)	60% (80% - 59%)	66% (62% - 74%)	90% (90% - 91%)
Widal (O) 1:80 (O)	53% (36% - 69%)	36% (69% - 86%)	29% (28% - 34%)	91% (91% - 92%)
Widal (O) 1:160 (O)	70% (51% - 85%)	51% (85% - 94%)	46% (41% - 55%)	96% (95% - 96%)
Widal (H) 1:160 (H)	30% (15% - 49%)	15% (49% - 98%)	19% (14% - 21%)	97% (96% - 97%)
Widal (O or H) 1:80 (O or H)	92% (75% - 99%)	75% (99% - 100%)	75% (73% - 76%)	99% (99% - 99%)
Widal (H) 1:200 (H), 1:100 (O)	30% (15% - 49%)	15% (49% - 91%)	19% (15% - 22%)	93% (91% - 94%)
Antibody (TPT test, S.Typhi specific IgA)	100% (94% - 100%)	94% (100% - 69%)	94% (76% - 100%)	97% (96% - 97%)
Widal (O) 1:180 (O)	61% (54% - 68%)	54% (68% - 51%)	67% (67% - 76%)	90% (87% - 97%)
Widal (H) 1:80 (H)	75% (48% - 93%)	48% (93% - 95%)	20% (15% - 28%)	97% (96% - 97%)

**Table 6:** Results from Bayesian latent class network meta-analysis in East-Asia and Pacific. Sensitivity and specificity in pediatric patients compared with a blood culture reference test or theoretical bone marrow culture test.

	Diagnostic accuracy against blood culture		Diagnostic accuracy against latent class bone marrow culture	
Test	Sensitivity (95%CI)	Specificity (95%CI)	Sensitivity (Scenario min-max)	Specificity (Scenario min-max)
ELISA IgG	47% (40% - 55%)	52% (49% - 55%)	54% (51% - 58%)	86% (81% - 89%)
ELISA IgM	63% (57% - 69%)	76% (74% - 79%)	65% (54% - 72%)	95% (92% - 99%)
TUBEX TP, O12	100% (88% - 100%)	100% (93% - 100%)	79% (54% - 99%)	99% (97% - 99%)
Lateral flow IgM	55% (39% - 70%)	98% (96% - 99%)	13% (10% -18%)	99% (97% - 100%)
PCR	41% (26% - 57%)	100% (99% - 100%)	7% (5% - 10%)	99% (97% - 100%)
Rapid Test	91% (59% - 100%)	96% (81% - 100%)	56% (42% - 78%)	99% (97% -99%)

**Table 7:** Results from Bayesian latent class network meta-analysis in Sub-Saharan Africa. Sensitivity and specificity in pediatric patients compared with a blood culture reference test or theoretical bone marrow culture test.

	Diagnostic accuracy against blood culture		Diagnostic accuracy against latent class bone marrow culture	
Test	Sensitivity (95%CI)	Specificity (95%CI)	Sensitivity (Scenario min-max)	Specificity (scenario min-max)
ELISA Total Ig	87% (74% - 95%)	75% (48% - 93%)	85% (81% - 88%)	92% (89% - 95%)
TUBEX IgM	79% (61% - 91%)	89% (81% - 94%)	47% (37% - 64%)	96% (94% - 98%)
PCR	88% (64% - 99%)	86% (80% - 90%)	34% (26% - 53%)	96% (95% - 99%)
Widal (O 1:80)	69% (41% - 89%)	96% (91% - 98%)	21% (15% - 30%)	99% (97% - 100%)
Widal (H 1:80)	75% (48% - 93%)	95% (90% - 98%)	18% (12% - 27%)	98% (97% - 100%)

**Table 8:** Results from Bayesian latent class network meta-analysis in South Asia. Sensitivity and specificity in pediatric patients compared with a blood culture reference test or theoretical bone marrow culture test.

	Diagnostic accuracy against blood culture		Diagnostic accuracy against latent class bone marrow culture	
Test	Sensitivity (95%CI)	Specificity (95%CI)	Sensitivity (Scenario min-max)	Specificity (Scenario min-max)
Enterocheck WB IgM	85% (73% - 93%)	89% (85% - 92%)	37% (28% - 54%)	97% (96% - 98%)
ELISA IgA	91% (79% - 98%)	79% (70% - 86%)	69% (53% - 83%)	86% (84% - 91%)
ELISA IgG	59% (45% - 72%)	71% (65% - 77%)	50% (44% - 54%)	74% (70% - 77%)
ELISA IgM	75% (62% - 86%)	78% (72% - 83%)	57% (48% - 64%)	91% (89% - 92%)
ELISA IgG or IgM	74% (56% - 87%)	80% (74% - 85%)	43% (35% - 58%)	91% (88% - 94%)
Lateral flow IgG	98% (90% - 99%)	78% (68% - 86%)	92% (72% - 98%)	94% (92% - 85%)
Lateral flow IgG or IgM	69% (57% - 79%)	71% (65% - 76%)	57% (47% - 65%)	89% (88% - 91%)
TUBEX IgM	63% (50% - 75%)	84% (81% - 88%)	44% (36% - 53%)	94% (91% - 97%)
Typhidot IgG	13% (4% - 30%)	33% (26% - 40%)	36% (21% - 48%)	66% (63% - 68%)
Typhidot IgM	77% (70% - 83%)	60% (56% - 65%)	75% (65% - 80%)	94% (90% - 98%)
Typhidot IgG or IgM	65% (44% - 83%)	66% (50% - 80%)	79% (76% - 91%)	90% (88% - 92%)
Reverse passive Hemagglutination	100% (93% - 100%)	76% (66% - 84%)	99% (98% - 100%)	84% (83% - 86%)
Diazo method	87% (69% - 96%)	86% (77% - 93%)	58% (45% - 74%)	96% (94% - 97%)
Nested PCR	44% (34% - 55%)	83% (78% - 88%)	39% (33% - 43%)	94% (92% - 95%)
PCR	48% (27% - 69%)	84% (76% - 90%)	33% (29% - 38%)	86% (81% - 89%)
TPT Test	100% (94% - 100%)	69% (62% - 75%)	90% (72% - 99%)	93% (91% - 94%)
Widal slide (O, 1:80)	71% (63% - 78%)	98% (93% - 100%)	60% (54% - 65%)	99% (99% - 100%)
Widal slide (H, 1:80)	86% (79% - 91%)	98% (93% - 100%)	76% (68% - 82%)	99% (99% - 100%)
Widal slide (O or H, 1:160)	74% (65% - 81%)	68% (62% - 74%)	66% (59% - 72%)	86% (82% - 88%)
Widal tube (O, 1:80)	57% (49% - 65%)	74% (66% - 80%)	54% (41% - 65%)	76% (72% - 77%)
Widal tube (O, 1:160)	70% (51% - 85%)	94% (83% - 99%)	48% (39% - 57%)	98% (97% - 99%)
Widal tube (H, 1:160)	30% (15% - 49%)	98% (89% - 100%)	18% (15% - 21%)	99% (98% - 99%)
Widal (O or H, 1:80)	92% (75% - 99%)	100% (29% - 100%)	72% (68% - 75%)	91% (90% - 94%)
Widal (O 1:100 or H 1:200)	30% (15% - 49%)	91% (83% - 96%)	19% (15% - 22%)	96% (94% - 97%)

Across all regions combined (Fig 3A and Table 5), rapid tests had both high sensitivity and specificity estimates. Among rapid tests, the reverse passive hemagluttination antigen test had 99% sensitivity (72% to 100% across scenario analyses) and 92% specificity; Typhidot IgM outperformed Typhidot IgG with 80% sensitivity (70% to 85% in scenario analyses) and 95% specificity; and Typhidot IgM or IgG had 91% sensitivity (86% to 93% in scenario analyses), however with specificity of 86%. ELISA IgM outperformed its IgG counterpart and the TPT test also performed very well with 94% sensitivity (76% to 100% in scenario analysis) and a specificity of 97%. The best Widal test appeared to be a 1:160 titer for the H-antigen slide test, yielding a sensitivity of 79% and a specificity of 98%. Lastly, the most sensitive test of all tests investigated for the pediatric population was the reverse passive hemagluttination antigen test however scenario analyses did yield fairly large model variability.

For EAP (Fig 3B and Table 6), the rapid test lateral flow IgM and PCR had very low sensitivity compared to the latent class bone marrow reference test (13% and 7% respectively). TUBEX TP, O12 was associated with a sensitivity of 79%, which was the highest among the investigated tests, and a specificity of 99%. ELISA IgG was inferior to ELISA IgM. The scenario analyses yielded modest sensitivity with ELISA IgM possibly yielding sensitivity up to 67%.

For Sub-Saharan Africa (Fig 3C and Table 7), ELISA Total Ig appeared superior to the other investigated tests with a sensitivity of 85% (81% to 88% in scenario analyses) and 92% specificity, which was the lowest specificity observed in the network analysis. Both Widal tests had very low sensitivity (<25% across all scenario analyses).

For South Asia (Fig 3D and Table 8), several rapid tests had both high sensitivity and specificity estimates. Among the rapid tests, the lateral-flow immunochromatographic dipstick IgG assay had 92% sensitivity (72% to 98% across scenario analyses) and 94% specificity; Typhidot IgM outperformed Typhidot IgG with 74% sensitivity (65% to 80% in scenario analyses) and 97% specificity; and Typhidot IgM or IgG had 79% sensitivity (76% to 91% in scenario analyses), however with specificity of 90%. ELISA IgM outperformed its IgG counterpart and the TPT test also performed very well with 90% sensitivity (72% to 99% in scenario analysis) and a specificity of 93%. The best Widal test appeared to be a 1:80 titer for the H-antigen slide test, yielding a sensitivity of 76% and a specificity of 99%. Lastly, the most sensitive test of all tests investigated for the South Asian pediatric population was Reverse Passive Hemagglutination with 99% sensitivity and scenario analyses did not yield large model variability.

Sensitivity and specificity of hypothetical combination tests are presented in Table 9 and were estimated for the South Asian population only, since none of the rapid tests in our subset of data were associated with good test performance characteristics in the two other World Bank regions. For acute care pediatric subjects tested in the South Asian setting, adding the ‘best’ Widal test (i.e., H-antigen slide test with cut-off 1:80) to any of the three highest performing rapid tests (reference tests: lateral flow IgG, TPT, and Typhidot IgM or IgG) did not yield marked improvements. Conversely, adding a lateral flow-based IgG rapid test to the TPT approach yielded improvements in sensitivity without substantial declines in specificity and was the best performing test combination.

**Table 9:** Combinations test estimates for South Asia. Sensitivity and specificity in pediatric patients compared with a theoretical bone marrow culture test.

	Diagnostic accuracy against latent class bone marrow	
Test	Sensitivity (95%CI)*	Specificity (95%CI)*
Widal slide H 1:80 or Lateral flow IgG	93% (73% -98%)	88% (86% - 89%)
Widal slide H 1:80 or Typhidot IgG or IgM	77% (72% -82%)	85% (85% - 88%)
Widal slide H 1:80 or TPT Test	89% (72% -99%)	87% (86% -88%)
Lateral flow IgG or Typhidot IgG or IgM	93% (76% - 98%)	90% (89% - 90%)
Lateral flow IgG or TPT Test	95% (78% - 99.8%)	92% (91% - 93%)
Typhidot IgG or IgM or TPT test	89% (75% - 99.9%)	89% (88% - 90%)

## Discussion

The results of this analysis builds the evidence base for typhoid diagnostics and is the first attempt to apply newly developed comparative methods for diagnostics testing accuracy.[16] This review and approach yielded several key insights. First, the body of studies on typhoid diagnostics and within study estimates of diagnostic accuracy were highly heterogeneous, even when restricting to studies with similar populations and study designs. Second, despite this heterogeneity, certain diagnostics consistently outperformed others; in particular, IgM-based ELISA and Typhidot outperformed their IgG-based counterparts, and the IgA-based TPT Test performed well in South Asia. Finally, the analytic methods allowed us to generate estimates for test performance based on combinations of tests. We found that combinations of existing sensitive and specific diagnostics may overcome the accuracy limitations inherent in single diagnostics, achieving what may be sufficient accuracy for use in certain clinical settings. Applying these methods allows us to generate estimates for test performance based on combinations of tests. This analysis has also provided comparative estimates of diagnostic testing accuracy for specific tests and targets across a more homogenous set of studies with similar age ranges, geographies and reference tests. This is an important addition because of the wide variety of test types within a family of targets such as antibody or antigen. Though there is an issue of regional variation in antibody response, the majority of our studies were from typhoid endemic regions likely with similar diagnostic titer cut-offs. This expanded and more detailed evidence base allows for more precise comparative assessments of diagnostic testing accuracy via indirect comparisons or network analysis.

The methods and results of this meta-analysis differ from previous meta-analyses of typhoid diagnostics, including those of Storey et al[9] and Wijedorou et al[51] in several ways. First, previous studies have focused on specific products rather than antigen/antibody combinations and performed single comparisons against a reference standard (a composite reference standard or blood culture), without performing between study comparisons through a network framework. We used latent class analysis to account for imperfect reference standards, which is critical given the low sensitivity of blood culture. Additionally, prior analyses focused on single diagnostics without examining their performance in combination and concluded that accuracy was insufficient. By focusing on diagnostic types and their combinations, and utilizing a network meta-analytic framework, we found that certain combinations of diagnostics exceeded 90% sensitivity and specificity.

Our analysis provides evidence that IgM-based ELISA and Typhidot assays diagnostics outperformed their IgG counterparts. Thriemer et al[14] performed a SLR and meta-analysis of the performance of Tubex TF and Typhidot in typhoid endemic countries and concluded that neither test was exclusively reliable for the diagnosis of the disease. Storey et al.[9] also concluded that no single test has sufficiently good performance but suggested that some existing diagnostics could be useful as part of a composite reference standard.

Our exploration of combination tests found, in the South Asian pediatric setting, combining a lateral flow IgG assay with the IgA-focused TPT test yields a high performing diagnostic combination. Combinations of the widely used Widal test and tests with good performance characteristics in Bayesian latent class analysis (lateral flow IgG or TPT test) did not yield substantial improvements to the individual tests alone.

We found that DNA-based tests, whether nested or not, performed similarly with limited sensitivity but high specificity. DNA diagnostic tests were few in our selected group of studies in children, likely due to the small blood volumes drawn from children and the need for substantial volumes for direct molecular diagnostics. The appeal of molecular diagnostics is that they can be more specific than serologies, more rapid than culture, and potentially less affected by prior antibiotic use. The main limitation is that the organism burden in blood during typhoid fever has been estimated at 0.1–1 CFU/ml[52]. For detection to be possible, a large volume of blood is needed, together with highly efficient DNA extraction, concentration and amplification. As a result, in practice, sensitivity is variable but often modest.

There are strengths and limitations to our analysis. Strengths include the extensive searching and identification of published and unpublished data. A further strength is the application of hierarchical modelling using the latent class analysis as it examines the strength of statistical relationships among variables. The analysis was also strengthened by our efforts to limit between-study heterogeneity through only including studies where: a reference test was included, the patient population consisted of children, and select geographical regions were examined. We assessed the potential for regional differences in diagnostic performance by dividing countries into World Bank regions; while these divisions are imperfect and the epidemiology may vary substantially within regions, there was not substantial variation in results in the NMA dataset, with few countries providing the majority of data. Our results were derived from data among children, who may be less likely to have prior exposure to typhoid and other infections compared with adults. It is possible that serologic cross reactivity to other pathogens may be more common in adults, and diagnostic accuracy may be lower. Therefore, we caution against extrapolating these findings to other age groups.

This study had several limitations. These were predominantly related to lack of studies in populations of interest to us. The majority of studies have been small, with over half of studies having less than 200 patients. In these studies–the risk of bias is high due to lack of statistical power and the higher chance of sampling bias. Furthermore, many of the studies were done using convenience sampling which leads to undefined study populations as whomever presented with index symptoms were included. Our results suggest there is a need for additional large sample studies of new methods/technologies to be confidently judged for their diagnostic accuracy. This echoes the conclusions of previous reviews and meta-analyses despite an enlarged and enhanced evidence base.[9] Further, in studies where a composite reference is used–there is a need for additional standardization of techniques and what constitutes a composite standard. In our attempt to extract specific data reference tests, different combinations of tests were used as the composite standard which complicates comparison across studies.

One of the challenges in summarizing evidence across diagnostic tests, such as serologic tests and molecular tests, is that very few studies used the same diagnostic approaches. The studies evaluating serologies used various combinations of antigens (e.g. Vi, Omp, LPS), antibody isotypes (IgG, IgM, IgA), and assay formats (commercial versus in-house ELISA, immunoblot, lateral flow), while studies evaluating molecular diagnostics used varying gene targets, extraction methods and PCR platforms. We therefore aggregated these diagnostics into “antibody”, “antigen” and “PCR” based tests to facilitate analysis of overall accuracy by general broad method; however, this precluded a more nuanced synthesis of evidence on which specific approaches and targets perform better.

A fundamental challenge with evaluating the accuracy of typhoid diagnostics is the lack of perfect reference standards. Bone marrow culture has the highest sensitivity, but was not used in most studies due to its invasiveness. Blood cultures, widely used due to their near perfect specificity, are only 50–65% sensitive. As a result, studies may inaccurately classify individuals with negative cultures as not having typhoid, which can in turn lead to under-estimates of the specificity of serologic diagnostics. To address this challenge and obtain comparative estimates of sensitivity and specificity with respect to bone marrow culture, we therefore applied a latent class extension to the conventional network meta-analysis model. The Bayesian framework allowed us to implement known diagnostics properties of bone marrow culture and the relationship between bone marrow and blood culture as informative priors to more accurately estimate the performance of various diagnostics.

Serologic tests for *S*. Typhi pose a particular challenge because, while surface antigens for typhoidal *Salmonella* are generally conserved, they are also shared with many other Enterobacteriaceae.[53] This means that diagnostic kits aimed at a general mix of *S*. Typhi antigens frequently suffer from low specificity.[53] Further the titres and specificities of antibodies to the classical typhoidal antigens O, H and Vi, vary a great deal, as demonstrated by studies of typhoidal antibody titres in endemic settings[54]. These issues pose challenges to the development of serologic assays built on these targets.

In conclusion, our analysis found a heterogeneous body of evidence for typhoid diagnostics. There is a high degree of variability in diagnostic testing characteristics across tests and regions even after restricting on patient population age, geographic region and reference test. Nevertheless, there are good combinations of existing tests that may provide opportunities in both for individual diagnosis as well as population-based surveillance. South Asia has the most informed set of data on typhoid diagnostic testing accuracy and the evidence base in other important regions needs to be expanded as the performance of diagnostics could vary by region and specific setting. In South Asia, there is evidence for good test performance of some rapid tests, but the evidence is variable due to limited numbers of studies once the data is stratified down by test type. Further work, particularly in the area of novel antigen detection, enhanced molecular diagnostic techniques, host transcriptional assays, metabolomic profiling and low-cost culture techniques all hold potential to drive real gains in the typhoid diagnostics space. Novel antigens specific for *S*. Typhi, as proposed by Baker et al[53], remains an exciting area of work given the variability of typhoid presentation. An important challenge would be the development of a panel of specific *S*. Typhi antigens that identify different stages of infection. These could be generated by testing cohorts of patients with protein microarrays in various specimen types to identify specific patterns of infection. Such studies, if fruitful, could lead to the development of low-cost assays. Novel culture techniques that are efficient and require minimal laboratory infrastructure would allow for improved burden estimation and a more accurate diagnosis, and therefore appropriate treatment.[55] To advance the evaluation of these new diagnostics, standardized clinical specimen biobanks representing multiple countries, populations and age groups should be established to facilitate direct comparison of multiple diagnostics against one another. Such a collaborative effort could help further overcome the limitations of population and diagnostic heterogeneity and imperfect reference standards that have limited diagnostic evaluation thus far, and accelerate the identification of accurate diagnostics for typhoid fever.

## Supporting information

S1 Table Search strategies for SLR. (XLSX)

S2 Table Data extraction form. (XLSB)

S3 Table Study level characteristics of 196 included studies. (XLSX)

S4 Table Comparison level data for 510 test comparisons. (XLSX)

S5 Table Glossary of terms. (XLSX)

S1 Checklist Preferred reporting items for systematic reviews and meta-analyses (PRISMA) 2009 checklist. (DOCX)

S1 Fig Forest plot of pair-wise meta-analysis of diagnostic test performance in East-Asia and Pacific. (TIF)

S2 Fig Forest plots of pair-wise meta-analysis of diagnostic test performance in sub-Saharan Africa. (TIF)

S3 Fig Forest plot of pair-wise meta-analysis of diagnostic test performance in South Asia part 1. (TIF)

S4 Fig Forest plot of pair-wise meta-analysis of diagnostic test performance in South Asia part 2. (TIF)

S1 Statistical appendix Methods summary of pairwise meta-analysis of diagnostic tests, model selection data for the network meta-analysis and OpenBUGS code for network meta-analysis of sub-Saharan Africa data. (DOCX)
