# Supplementary figures and images for: Comparative accuracy of typhoid diagnostic tools: A Bayesian latent-class network analysis
Source: PLoS Negl Trop Dis. 2019 May 8;13(5):e0007303. doi: 10.1371/journal.pntd.0007303 (PMC6527309; doi:10.1371/journal.pntd.0007303)

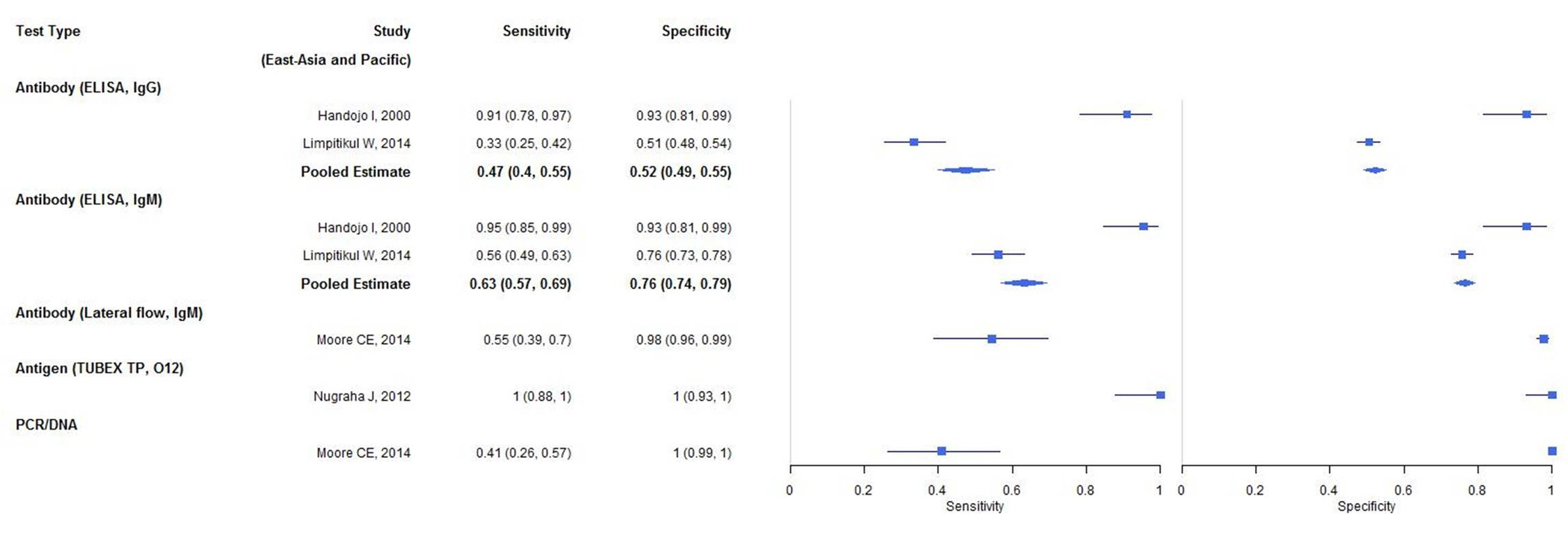

Supplement: S1 Fig — (TIF) [file pntd.0007303.s007.tif]

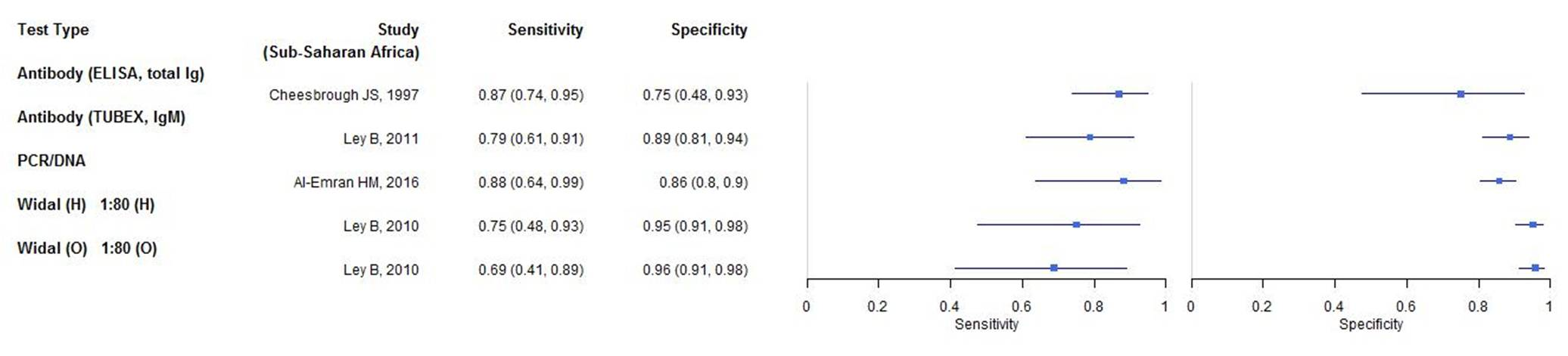

Supplement: S2 Fig — (TIF) [file pntd.0007303.s008.tif]

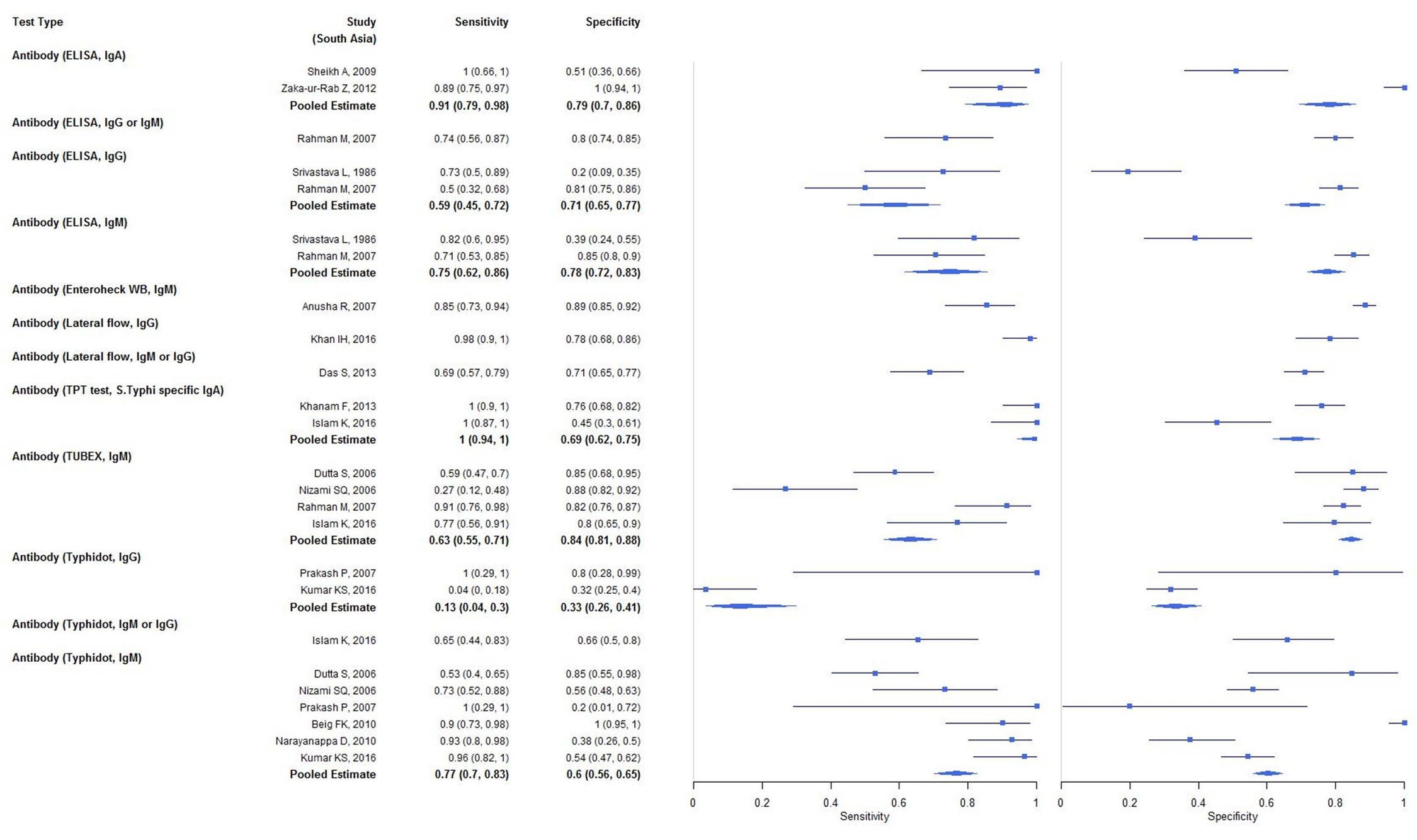

Supplement: S3 Fig — (TIF) [file pntd.0007303.s009.tif]

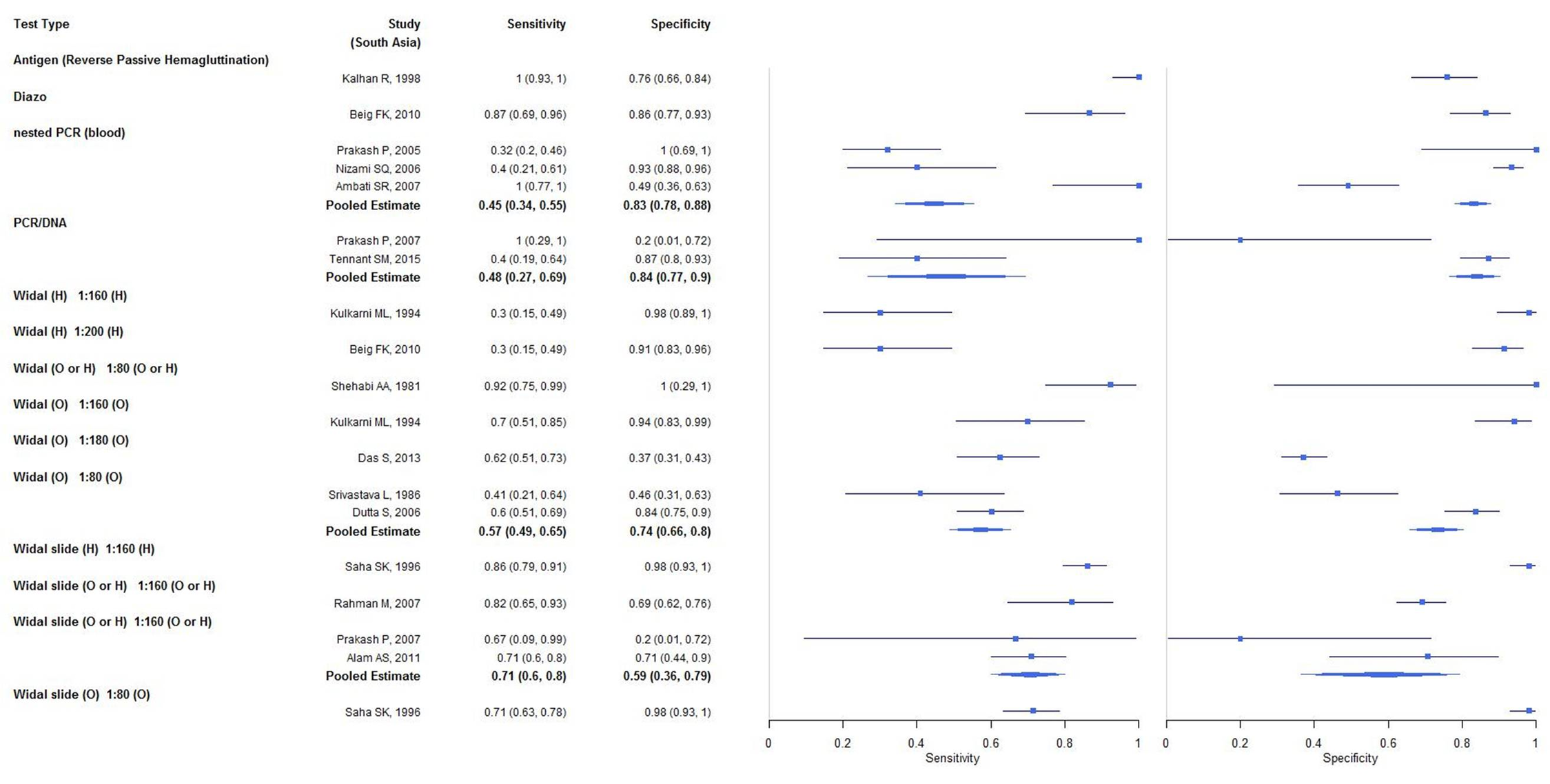

Supplement: S4 Fig — (TIF) [file pntd.0007303.s010.tif]
