# Supplementary material for: Comparative accuracy of typhoid diagnostic tools: A Bayesian latent-class network analysis
Source: PLoS Negl Trop Dis. 2019 May 8;13(5):e0007303. doi: 10.1371/journal.pntd.0007303 (PMC6527309; doi:10.1371/journal.pntd.0007303)
Supplement: S1 Statistical appendix — (DOCX) [file pntd.0007303.s011.docx]

**Statistical Appendix: Methods summary of pairwise meta-analysis of diagnostic tests, model selection data for the network meta-analysis and OpenBUGS code for network meta-analysis of Sub-Saharan Africa data.**

**Pairwise meta-analysis of diagnostic tests**

As described by Harbord and Whiting, the bivariate model developed by Reitsma et al models the sensitivity and specificity assuming that a log odds (logit) transformation of each has a bivariate normal distribution across studies. Transformed sensitivities are assumed to have a mean of μA and a variance of σ2A and the logit-transformed specificities have a mean of μB and a variance of σ2B. The relationship between sensitivity and specificity is allowed for by including a correlation, ρAB, that has a negative expectation.

**Network meta-analysis model**

Assume that the number of true positives, *N_TP_*, and the number of true negatives, *N_TP_*, in a study follow the binomial distributions *N_TP_ ~ bin(N_D_ , S)* and *N_TN_ ~ bin(N_ND_ , C)*, where *N_D_* and *N_ND_* are the number of diseased and non-diseased, respectively, and where *S* and *C* are the sensitivity and specificity of the test in the study population, respectively. We can then model the sensitivity and specificity using a bivariate logistic model. In particular, by letting *g()* denote the logistic link function and letting *θ_S_ = g(S)* and *θ_C_ = g(C)*, we model *θ_S_* and *θ_C_* as a bivariate normal distribution with mean (*μ_S_* , *μ_C_)* and covariance matrix Σ. In the context of meta-analysis, the *θ’s* are modelled across studies *i* and tests *j* and the *μ’s* represent the diagnostic accuracies by each test *j*. However, this approach simply pools sensitivity and specificity results, and does not account for the need to pool across comparative accuracy between tests. Rather, the model can be expanded to anchor in the reference test, with logit transformed sensitivities and specificities represented as *μ’s*. Accordingly, the index test can be represented as a combination of the *μ’s* an the comparative diagnostic accuracy between the index test and the reference test, represented as δ. Thus, with 1 subscript denoting the reference test, 2 subscript denoting the index test, and *i* subscript denoting the study number, we can mathematically represent any comparison between two test for each study as follows:

*θ_Si1_ = μ_Si_*

*θ_Si2_ = μ_Si_ +* δ*_Si_*

*θ_Ci1_ = μ_Ci_*

*θ_Ci2_ = μ_Ci_ +* δ*_Ci_*

Where δ_S_ is the comparative sensitivity log odds ratio between the tests, and δ_C_ is the comparative specificity log odds ratio between the tests. We then model δ_S_ and δ_C_ as a bivariate normal distribution with mean (*ν_S_* , *ν_C_)* and covariance matrix Σ. When multiple tests are included in the analyses this equation simply expands as follows:

*θ_Si1_ = μ_Si_*

*θ_Si2_ = μ_Si_ +* δ*_Si2_*

$$\vdots$$

*θ_SiK_ = μ_Si_ +* δ*_SiK_*

*θ_Ci1_ = μ_Ci_*

*θ_Ci2_ = μ_Ci_ +* δ*_Ci2_*

$$\vdots$$

*θ_CiK_ = μ_Ci_ +* δ*_CiK_*

Where the added subscript denotes the test (for tests 1, 2,…K). In addition, for any two index tests M and N, the difference between these two tests for sensitivity and specificity, respectively, are modelled as *ν_SN_ -* *ν_SM_* and *ν_CN_-* *ν_CM_,* to ensure consistency between direct and indirect estimates.

As network meta-analyses are conventionally performed in the Bayesian framework, vague priors are typically assigned to the modelled parameters as described elsewhere (Menten and Lesaffre). When the reference test is not a perfect gold standard, one can apply a Bayesian latent class approach in which informative prior distributions are applied to create the latent class gold standard from the imperfect reference standard. In the case of typhoid, it is generally known that blood culture is only 50% sensitive, but 100% specific. Thus, an informative prior to resample some of the observed negative test outcomes to become positive can be applied. In particular, a Bernoulli prior with fixed probability of resampling a negative value to become negative can be applied. Further, it is important to specify the assumed sensitivity and specificity of the gold standard if known. For typhoid disease, the gold standard is bone marrow culture and its diagnostic accuracy is generally well-known to be 85-95% sensitive and 100% specific. Thus, informative normal priors can be applied to the *μ_Si_* and *μ_Ci_* parameters. For example, assuming 95% sensitivity, the corresponding log odds ratio of ln(0.95/0.05)=2.94 can be chosen as the mean of informative prior accompanied by sufficiently high precision.

**OpenBUGS code for network meta-analysis of Sub-Saharan Africa data (fixed-effect example).**

model{ #model begins

# for loop likelihood for individual patient data points

for (i in 1:nPats){ #nPats is the total number of individual patients

# reference standard for disease status

# samples % negative blood samples that would be positive with bone marrow

# base case: 0.5, scenario analyses: 0.333 and 0.667

PC[i] ~ dbern(0.5)
 status[i] <- max(RS[i],PC[i])

# Results for each test follow independent Bernoulli distributions,

# variable Y[i] with probability determined by the study-specific,

# logit-transformed sensitivity (alpha) and specificity (beta)

Y[i] ~ dbern(P[i])

logit(P[i]) <- status[i] * alpha[Comp[i],t[i]] + (1-status[i]) *

beta[Comp[i],t[i]]

} # for loop likelihood for individual patient data ends

# Model for study-specific, logit-transformed sensitivity (alpha)

# and specificity (beta)

# mu.site[j,1] is the average (logit) probability of testing positive with # the index test in the diseased in site j

# mu.site[j,2] is the average (logit) probability of testing positive with # the index test in the non-diseased in site j

for(j in 1:nComp){ #nComp is the tocal number of comparisons

# Informative prior on bone marrow sensitivity

mu.site[j,1] ~ dnorm(2.94,5)I(1,) # base case 95%

# mu.site[j,1] ~ dnorm(1.73,5)I(1,) # scenario analysis 85%

# Informative prior on bone marrow specificity

mu.site[j,2] ~ dnorm(-6.91,5)I(,-1) # base case 99.9%

# mu.site[j,2] ~ dnorm(-4.60,5)I(,-1) # scenario analysis 99%

alpha[j,1] <- mu.site[j,1]

beta[j,1] <- mu.site[j,2]

alpha[j,ts[j]] <- mu.site[j,1]+d.a[ts[j]]

beta[j,ts[j]] <- mu.site[j,2] +d.b[ts[j]]

}

### Priors for study level results ###

## Means of contrasts between (logit) sensitivity (d.a) and specificity (d.b)

d.a[1]<-0

d.b[1]<-0

for(k in 2:(nt+1)){

d.a[k] ~ dnorm(0.0,0.001)I(-6,6)

d.b[k] ~ dnorm(5,1)I(1,) # semi-informative prior to stabilize specificity

}

# Obtain estimates for summary statistics average logit probability

# of testing positive (mu[1]) and negative (mu[2])

mu[1] <- mean(mu.site[,1])

mu[2] <- mean(mu.site[,2])

# Converting to sensitivity and specificity

logit(SENS[1]) <- mu[1]

logit(SPEC[1] )<- 1-mu[2]

for(i in 2:(nt+1)){

logit(SENS[i]) <- mu[1]+d.a[i]

logit(SPEC[i]) <- 1-(mu[2]+d.b[i])

}

# estimating sensitivities and specificities for hypothetical combination therapies

# Sensitivity

SENS[26] <- max(SENS[7],SENS[19]) # Widal slide H 1:80 or Lateral flow IgG

SENS[27] <- max(SENS[12],SENS[19]) # Widal slide H 1:80 or Typhidot IgG or IgM

SENS[29] <- max(SENS[25],SENS[19]) # Widal slide H 1:80 or TPT Test

SENS[30] <- max(SENS[12],SENS[7]) # Lateral flow IgG or Typhidot IgG or IgM

SENS[31] <- max(SENS[25],SENS[7]) # Lateral flow IgG or TPT Test

SENS[32] <- max(SENS[25],SENS[12]) # Typhidot IgG or IgM or TPT Test

# Specificity

SPEC[26] <- min(SPEC[7],SPEC[19]) # Widal slide H 1:80 or Lateral flow IgG

SPEC[27] <- min(SPEC[12],SPEC[19]) # Widal slide H 1:80 or Typhidot IgG or IgM

SPEC[28] <- min(SPEC[25],SPEC[19]) # Widal slide H 1:80 or TPT Test

SPEC[29] <- min(SPEC[12],SPEC[7]) # Lateral flow IgG or Typhidot IgG or IgM

SPEC[30] <- min(SPEC[25],SPEC[7]) # Lateral flow IgG or TPT Test

SPEC[31] <- min(SPEC[25],SPEC[12]) # Typhidot IgG or IgM or TPT Test

} # model ends
